# Differential production of superoxide by neuronal mitochondria

**DOI:** 10.1186/1471-2202-9-4

**Published:** 2008-01-08

**Authors:** Mark J Hoegger, Christopher J Lieven, Leonard A Levin

**Affiliations:** 1 Department of Ophthalmology and Visual Sciences, University of Wisconsin Medical School, Madison, USA; 2 Department of Ophthalmology, University of Montreal and Maisonneuve-Rosemont Hospital, Montreal, Canada

## Abstract

**Background:**

Mitochondrial DNA (mtDNA) mutations, which are present in all mitochondria-containing cells, paradoxically cause tissue-specific disease. For example, Leber's hereditary optic neuropathy (LHON) results from one of three point mutations mtDNA coding for complex I components, but is only manifested in retinal ganglion cells (RGCs), a central neuron contained within the retina. Given that RGCs use superoxide for intracellular signaling after axotomy, and that LHON mutations increase superoxide levels in non-RGC transmitochondrial cybrids, we hypothesized that RGCs regulate superoxide levels differently than other neuronal cells. To study this, we compared superoxide production and mitochondrial electron transport chain (METC) components in isolated RGC mitochondria to mitochondria isolated from cerebral cortex and neuroblastoma SK-N-AS cells.

**Results:**

In the presence of the complex I substrate glutamate/malate or the complex II substrate succinate, the rate of superoxide production in RGC-5 cells was significantly lower than cerebral or neuroblastoma cells. Cerebral but not RGC-5 or neuroblastoma cells increased superoxide production in response to the complex I inhibitor rotenone, while neuroblastoma but not cerebral or RGC-5 cells dramatically decreased superoxide production in response to the complex III inhibitor antimycin A. Immunoblotting and real-time quantitative PCR of METC components demonstrated different patterns of expression among the three different sources of neuronal mitochondria.

**Conclusion:**

RGC-5 mitochondria produce superoxide at significantly lower rates than cerebral and neuroblastoma mitochondria, most likely as a result of differential expression of complex I components. Diversity in METC component expression and function could explain tissue specificity in diseases associated with inherited mtDNA abnormalities.

## Background

Maternally inherited mitochondrial DNA (mtDNA) codes for several mitochondrial proteins, primarily components of the mitochondrial electron transport chain (METC). Point mutations in mtDNA are causes of several diseases, including mitochondrial encephalomyopathy with lactic acidosis and stroke-like episodes (MELAS; position 3243 and others); neuropathy, ataxia, and retinitis pigmentosa (NARP; position 8993); and Leber's hereditary optic neuropathy (LHON; positions 11778, 3460, and 14484). LHON is the most common hereditary optic nerve disease causing severe visual loss. Most primary mutations code for proteins in METC complex I, although some secondary mutations are in complex III [1]. A fundamental paradox of diseases caused by mtDNA point mutations is that although all mitochondria in the body have the same mutation (when homoplasmic), the disease is usually expressed in a restricted number of tissues. In the case of LHON, the disease is almost exclusively manifested in retinal ganglion cells (RGCs), the axons of which make up the optic nerve. The reason for this cell-type specificity is unknown, but presumably is related to a particular susceptibility of RGCs to a consequence of these mutations.

The effect of LHON mutations has been studied by producing transmitochondrial cybrids in human cell lines, demonstrating upregulation of some mtDNA transcripts, e.g. aldose reductase [2]. LHON cybrids forced to use the METC as their primary energy source by plating on glucose-deficient galactose media, experience apoptotic death and increased cytochrome c release [3]. Common LHON mutations reduce the rate of respiration in LHON cybrids and oxygen consumption [4,5]. Most importantly, LHON cybrids have increased superoxide production compared to wild-type cells [6].

Given that METC complexes I and III are the main sources of basal superoxide production [7,8], it is possible that aberrant production of superoxide from mutated METC components would cause cell death. But why would abnormalities in superoxide production be a mechanism for the relatively specific effect of LHON mtDNA mutations on RGCs? One explanation is the finding that RGCs use superoxide as an intracellular signal for initiating the apoptosis program after apoptosis [9,10], probably by oxidizing critical sulfhydryls in signaling macromolecules [11,12]. Supporting this theory, RGC death is triggered when mitochondrial superoxide levels are increased by knocking down mitochondrial superoxide dismutase (SOD-2) [13]. Given that RGCs use superoxide for intracellular signaling, and that they are specifically affected in a disease of specific mtDNA mutations, we examined superoxide production in isolated RGC mitochondria, using the RGC-5 RGC cell line. We show that mitochondria derived from RGCs differ from cerebral and neuroblastoma mitochondria with respect to superoxide production in the presence of complex-specific METC substrates and inhibitors. Furthermore, we showed distinct patterns of METC component expression. Differences in superoxide production between neuronal cell types could prevent aberrant apoptosis signaling [10], and its disturbance with mtDNA mutations [6] could account for tissue-specific expression of disease phenotype.

## Results

### Mitochondria can be isolated in bulk from RGC-5 cells

**Figure 1:**
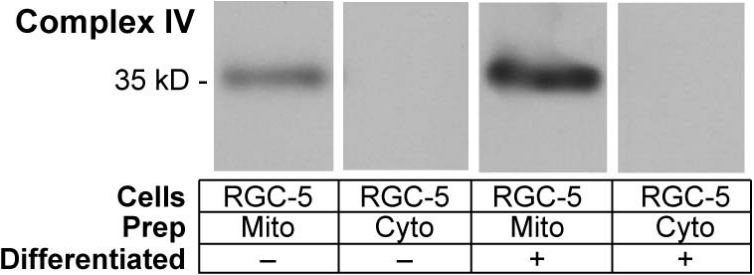
**Immunoblotting Measurement of Cytochrome c Oxidase in Differentiated and Undifferentiated RGC-5 Mitochondria**. RGC-5 cells were differentiated with staurosporine. Isolated mitochondrial samples from undifferentiated and differentiated mitochondria standardized for protein content were compared to corresponding mitochondria depleted samples for the presence of the mitochondrial transmembrane protein cytochrome c oxidase (COX), complex IV. Samples were immunoblotted with antibody to subunit 1 of complex IV. There was considerable purification of mitochondria compared to the mitochondria depleted samples for differentiated and undifferentiated RGC-5 cells. Differentiated RGC-5 cells had 2.5 times more cytochrome c oxidase than undifferentiated RGC-5 cells.

**Figure 2:**
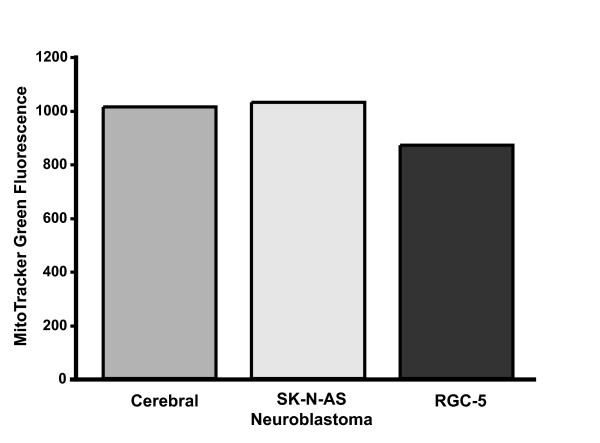
**Mitochondrial Quantification by MitoTracker Green**. Isolated mitochondria preparations were corrected for protein concentration and then treated with MitoTracker Green, which becomes fluorescent in the lipid environment of active mitochondria. Fluorescence is in arbitrary units. Mitochondrial samples from RGC-5 cells, neuroblastoma cells, and cerebral cells exhibited similar fluorescence values, indicating similar amounts of mitochondria per unit protein.

Because RGCs are post-mitotic and are present in relatively small (10^5 ^cells/retina in the rat) numbers, it is impractical to biochemically study RGC mitochondria in bulk. Instead, we used the RGC-5 cell line, which when differentiated are phenotypically similar to RGCs [14]. RGC-5 cells were grown in tissue culture and mitochondria isolated. To assess purity of mitochondrial isolation, mitochondrial-enriched and cytosolic fractions were immunoblotted for cytochrome c oxidase, demonstrating significant enrichment (Figure 1). Degree of mitochondrial purification was similar among cell-types, based on quantitation with the fluorophore MitoTracker Green FM, which reacts with mitochondrial free sulfhydryls. Mitochondrial samples exhibited similar relative fluorescence values per mg protein (Figure 2).

### Cerebral and RGC-5 cells differ in superoxide production with complex I substrates and after complex I inhibition

**Figure 3:**
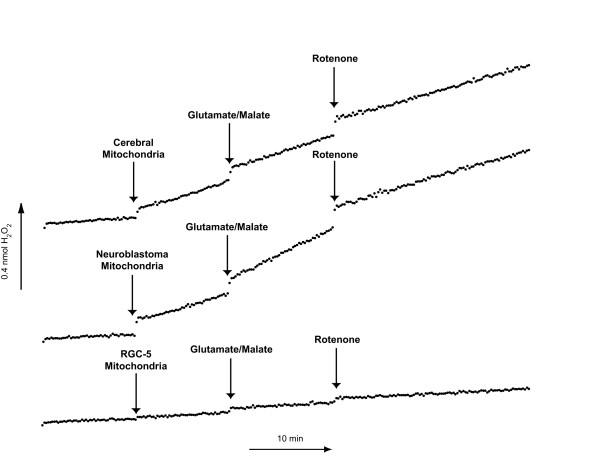
**Superoxide Production in Cerebral, Neuroblastoma, and RGC-5 Mitochondria after Complex I Inhibition**. Mitochondria isolated from cerebral, neuroblastoma, and undifferentiated RGC-5 cells were standardized for protein content and 50 μl were added to a well in a 96-well plate containing 50 μL Amplex Red (AR). Prior to the addition of solutions containing mitochondria a baseline of superoxide production was established by measuring the fluorescent product of H_2_O_2 _and AR, resorufin at approximately 10-second intervals. In a similar manner, production of superoxide was analyzed after the addition of the complex I substrates glutamate/malate and the complex I inhibitor rotenone. The mitochondrial superoxide production levels after the addition of glutamate/malate in cerebral cells were significantly higher than that of undifferentiated RGC-5 cells during basal metabolism, in the presence of glutamate/malate and after complex I inhibition with rotenone.

Superoxide was indirectly measured by detecting H_2_O_2 _generation over time as a result of spontaneous dismutation of superoxide by mitochondrial SOD-2 [15,16]. All results are expressed as the mean ± SEM (in nmol/min/mg protein) of 5 independent experiments, each in duplicate. A typical experiment is depicted in Figure 3. The basal level of superoxide production by RGC-5 mitochondria in the absence of substrate was 0.030 ± 0.004 nmol/min/mg protein, approximately one-seventh that of cerebral mitochondria (0.208 ± 0.033; p = 0.0017). Mitochondria were then incubated with glutamate (10 mM) and malate (5 mM), which yields NADH and serves as a substrate for complex I. In the presence of glutamate/malate there was a small but significant increase in superoxide production in cerebral (0.262 ± 0.028; p = 0.0042 compared to no substrate) and RGC-5 mitochondria (0.046 ± 0.004; p = 0.0005 compared to no substrate). When samples were then treated with the complex I inhibitor rotenone (6.7 μM) there was an insignificant change in the rate of superoxide production in both cerebral mitochondria (0.279 ± 0.035; p = 0.40 compared to glutamate/malate alone) and RGC-5 mitochondria (0.048 ± 0.004; p = 0.24) after the addition of rotenone. Nonetheless the rates of superoxide production per mg protein in all conditions were significantly lower in RGC-5 mitochondria than in cerebral mitochondria (Table 1).

**Table 1:** Superoxide Production in Mitochondria from Cerebral, Neuroblastoma, and Undifferentiated RGC-5 Cells in the Presence of a Complex I Substrate and Inhibitor.

	**Cerebral**		**Neuroblastoma**		**RGC-5**	
	**N = 7**		**N = 7**		**N = 8**	

	**Mean**	**SEM**	**Mean**	**SEM**	**Mean**	**SEM**

**Mitochondria**	0.208	0.033	0.318	0.035	0.030	0.004
**Glutamate/Malate**	0.262	0.028	0.560	0.054	0.046	0.004
**Rotenone**	0.279	0.035	0.383	0.050	0.048	0.004

The rates of superoxide production were determined after the addition of the complex I substrates glutamate (10 mM) and malate (5 mM) and the complex I inhibitor rotenone (6.7 μM). Superoxide was indirectly measured using the H_2_O_2 _probe Amplex Red in the presence of horseradish peroxidase (HRP). The values are given in nmol H_2_O_2_/min/mg protein ± standard error of the mean (SEM), with n being the number of independent samples for each mitochondria type.

### Cerebral and RGC-5 mitochondria differ in superoxide production with complex II substrate and after complex III inhibition

**Figure 4:**
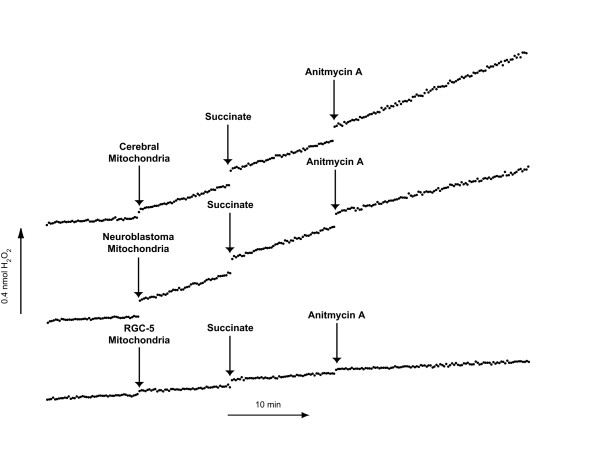
**Superoxide Production in Cerebral, Neuroblastoma, and RGC-5 Mitochondria after Complex III inhibition**. Mitochondria isolated from cerebral, neuroblastoma, and undifferentiated RGC-5 cells were normalized for protein content and superoxide production was analyzed after the addition of the complex II substrate succinate and the complex III inhibitor antimycin A. The mitochondrial superoxide production levels in cerebral cells were significantly higher than that of undifferentiated RGC-5 cells in the presence of succinate and after complex III inhibition with antimycin A. Both succinate and antimycin A elicited minimal superoxide production in undifferentiated RGC-5 cells, while in cerebral cells there was a near two-fold increase in superoxide production after the addition of antimycin A.

Mitochondria were incubated with succinate (10 mM), which yields FADH_2 _and serves as a substrate for complex II. In the presence of succinate, there was a small but significant increase in superoxide production in cerebral mitochondria (0.193 ± 0.027 to 0.216 ± 0.022; p = 0.04) and RGC-5 mitochondria (0.028 ± 0.005 to 0.031 ± 0.005; p = 0.024). Subsequent treatment with the complex III inhibitor antimycin A (0.5 μM) resulted in a dramatic increase in the rate of superoxide production in cerebral mitochondria (0.540 ± 0.116; p = .023 compared to substrate alone), but not RGC-5 mitochondria (0.024 ± 0.004; p = 0.278). The rates of superoxide production in all conditions were substantially greater in cerebral mitochondria than in RGC-5 mitochondria (Table 2). A typical experiment is depicted in Figure 4.

**Table 2:** Superoxide Production in Mitochondria from Cerebral, Neuroblastoma, and Undifferentiated RGC-5 Cells in the Presence of a Complex II Substrate and a Complex III Inhibitor.

	**Cerebral**		**Neuroblastoma**		**RGC-5**	
	**N = 7**		**N = 7**		**N = 8**	

	**Mean**	**SEM**	**Mean**	**SEM**	**Mean**	**SEM**

**Mitochondria**	0.193	0.027	0.348	0.042	0.028	0.005
**Succinate**	0.216	0.022	0.307	0.024	0.031	0.005
**Antimycin A**	0.540	0.116	0.277	0.034	0.024	0.004

The rates of superoxide production were determined after the addition of the complex II substrate succinate (10 mM) and the complex III inhibitor antimycin A (0.5 μM). Superoxide was indirectly measured using the H_2_O_2 _probe Amplex Red in the presence of horseradish peroxidase (HRP). The values are given in nmol H_2_O_2_/min/mg protein ± standard error of the mean (SEM), with n being the number of independent samples for each mitochondria type.

### Differentiation of RGC-5 cells does not normalize mitochondrial superoxide production

One explanation for the differences in mitochondrial superoxide production between cerebral and RGC-5 cells is the fact that RGC-5 cells are mitotic, while cerebral neurons are post-mitotic. To address the possibility that differences in proliferative state, metabolic activity, or degree of differentiation affected superoxide production by mitochondria, we differentiated RGC-5 cells with the broad spectrum kinase inhibitor staurosporine, which we have previously shown to induce a RGC phenotype without inducing apoptosis [14]. We then measured superoxide production rates as above (Tables 3 and 4).

**Table 3:** Superoxide Production in Mitochondria from Differentiated and Undifferentiated RGC-5 Cells in the Presence of a Complex I Substrate and Inhibitor.

	**Undifferentiated RGC-5**		**Differentiated RGC-5**	
	**N = 8**		**N = 2**	

	**Mean**	**SEM**	**Mean**	**SEM**

**Mitochondria**	0.030	0.004	0.033	0.004
**Glutamate/Malate**	0.046	0.004	0.052	0.003
**Rotenone**	0.048	0.004	0.055	0.003

The rates of superoxide production were determined after the addition of the complex I substrates glutamate (10 mM) and malate (5 mM) and the complex I inhibitor rotenone (6.7 μM). Superoxide was indirectly measured using the H_2_O_2 _probe Amplex Red in the presence of horseradish peroxidase (HRP). The values are given in nmol H_2_O_2_/min/mg protein ± standard error of the mean (SEM), with n being the number of independent samples for each mitochondria type.

**Table 4:** Superoxide Production in Mitochondria from Differentiated and Undifferentiated RGC-5 Cells in the Presence of a Complex II Substrate and a Complex III Inhibitor.

	**Undifferentiated RGC-5**		**Differentiated RGC-5**	
	**N = 8**		**N = 2**	

	**Mean**	**SEM**	**Mean**	**SEM**

**Mitochondria**	0.028	0.005	0.041	0.002
**Succinate**	0.031	0.005	0.037	0.001
**Antimycin A**	0.024	0.004	0.040	0.002

The rates of superoxide production were determined after the addition of the complex II substrate succinate (10 mM) and the complex III inhibitor antimycin A (0.5 μM). Superoxide was indirectly measured using the H_2_O_2 _probe Amplex Red in the presence of horseradish peroxidase (HRP). The values are given in nmol H_2_O_2_/min/mg protein ± standard error of the mean (SEM), with n being the number of independent samples for each mitochondria type.

Basal mitochondrial superoxide production was similar in undifferentiated and differentiated RGC-5 cells (0.030 ± 0.004 vs. 0.033 ± 0.004; p = 0.60). Mitochondria from undifferentiated and differentiated RGC-5 were incubated with glutamate/malate and subsequently treated with rotenone. There was no significant difference between differentiated and undifferentiated RGC-5 cells in the production of superoxide after treatment with glutamate/malate (0.046 ± 0.004 vs. 0.052 ± 0.003; p = 0.30) or rotenone (0.048 ± 0.004 vs. 0.055 ± 0.002; p = 0.18). Mitochondria from undifferentiated and differentiated RGC-5 did not significantly differ in rates of superoxide production when incubated with the complex II substrate succinate (0.031 ± 0.005 vs. 0.037 ± 0.001; p = 0.30). However, addition of antimycin A resulted in somewhat more superoxide production in differentiated but not undifferentiated RGC-5 cells (0.040 ± 0.002 vs. 0.024 ± 0.004; p = 0.001). Nonetheless, mitochondria from differentiated RGC-5 cells had much lower superoxide production than cerebral mitochondria under all treatment conditions.

### RGC-5 cells generate significantly less superoxide than neuroblastoma SK-N-AS cells with complex I and complex II substrates

The differences between superoxide generation from RGC-5 and cerebral mitochondria could theoretically reflect differences in the source of cells, a cultured cell line in the former and fresh tissue in the latter. To rule out this possibility, we compared superoxide generation in RGC-5 mitochondria to another neuronal cell line, the SK-N-AS neuroblastoma line (Figures 3 and 4, Tables 1 and 2). As with cerebral cells, the basal superoxide production was much lower in RGC-5 cells compared to neuroblastoma cells (0.030 ± 0.004 vs. 0.318 ± 0.035; p = 0.0002). Superoxide generation from RGC-5 and SK-N-AS cells was measured after the addition of glutamate/malate. There was a large increase in superoxide production after the addition of glutamate/malate to SK-N-AS mitochondria (0.560 ± 0.054; p < 0.0001 compared to no substrate), similar to what was seen with cerebral and RGC-5 mitochondria. After the addition of rotenone there was a significant decrease in superoxide production in SK-N-AS cells (0.383 ± 0.050; p < 0.0001 compared to substrate alone), unlike what was seen with cerebral or RGC-5 cells. In experiments where the complex II substrate was added to neuroblastoma mitochondria, there was a minimal change in production of superoxide (0.348 ± 0.042 to 0.307 ± 0.024; p = 0.08), similar to cerebral and RGC-5 mitochondria. The addition of antimycin A to neuroblastoma mitochondria resulted in a small but significant decrease in superoxide production (0.277 ± 0.034; p = 0.026 compared to substrate alone), similar to RGC-5 mitochondria but different from the increase in superoxide seen in cerebral mitochondria. In all cases, the rates of superoxide production by neuroblastoma mitochondria were much higher than RGC-5 mitochondria.

### Mitochondria isolated from RGC-5, neuroblastoma SK-N-AS, and cerebrum differ in content of METC components

**Figure 5:**
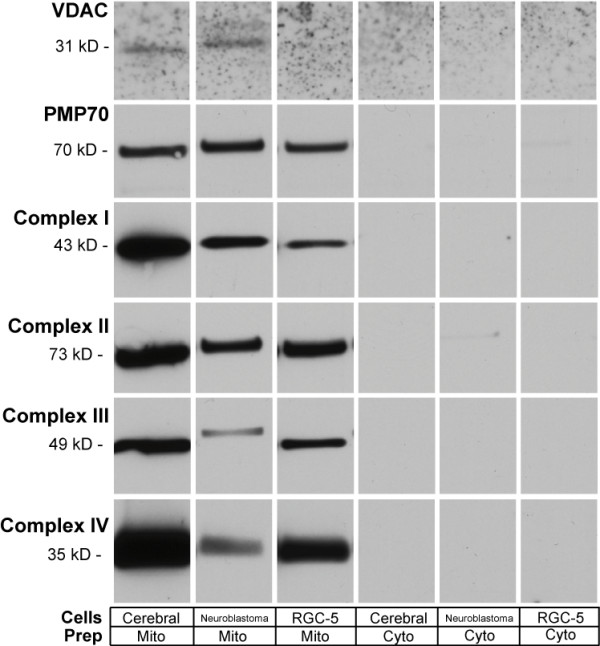
**Immunoblotting of Mitochondrial Components**. Isolated mitochondrial samples standardized for protein content were compared to corresponding mitochondria depleted samples for the presence of the mitochondrial membrane proteins Complexes I-IV, and VDAC. Standardized mitochondrial preparations were also investigated for the peroxisomal marker PMP70. Samples were subject to an electrophoresis and were transferred overnight to nitrocellulose. Samples were incubated with a mouse a primary antibody corresponding the protein of interest, and subsequently treated with an HRP-conjugated antibody. Each mitochondrial protein shows that there is considerable purification of mitochondria compared to the mitochondria depleted samples for cerebral, neuroblastoma, and undifferentiated RGC-5 cells. There is a nearly 18-fold increase in the expression of complex I in cerebral cells compared to RGC-5. Similar levels of expression in PMP70 and complex II were seen in all three samples.

Most mitochondrial superoxide is produced by the METC. To determine if differences in superoxide production among mitochondria from different neuronal subtypes could be caused by differences in the METC, we measured the relative amounts of METC complexes for protein concentration-corrected mitochondrial isolates. Immunoblots against specific METC complex subunits were scanned by densitometry and normalized to concentrations from cerebral mitochondria (Figure 5 and Table 5). The most surprising finding was that RGC-5 mitochondria contained only one-sixteenth as much METC complex I as was present in cerebral cells. Complex II, III, and IV components were expressed at a molar concentration in RGC-5 mitochondria about half that of cerebral mitochondria. Neuroblastoma mitochondria had similar levels of complexes I-IV, ranging from 16% to 46% that of brain.

**Table 5:** Density Comparison of METC Components in Cerebral, Neuroblastoma, and Undifferentiated RGC-5 Mitochondrial Samples.

	**Complex I**	**Complex II**	**Complex III**	**Complex IV**	**VDAC**	**PMP70**
**Cerebral**	1	1	1	1	1	1
**Neuro-blastoma**	0.258	0.456	0.158	0.224	1.625	1.032
**RGC-5**	0.056	0.654	0.533	0.561	0.599	0.797

The band density of mitochondrial related proteins were compared between cerebral, neuroblastoma, and RGC-5 derived mitochondrial samples for METC complexes I-IV. A comparison of VDAC and PMP70 expression were used to determine the presence of non-METC mitochondrial components and peroxisomal contamination, respectively. Cerebral, RGC-5, and neuroblastoma values are given as ratios compared to the band density of cerebral mitochondria.

We considered the possibility that different physical properties of cells or mitochondria might produce differential purification of mitochondria. The primary organelles co-purified with mitochondria are peroxisomes. To investigate the possibility that peroxisomal contamination could decrease the number of mitochondria with the preparations, we immunoblotted protein-corrected mitochondrial isolates for the peroxisomal protein PMP70. There were similar amounts of PMP70 in the mitochondria purified from RGC-5 cells, neuroblastoma cells, and cerebral cells (Figure 5 and Table 5), ruling out differential peroxisomal contamination as the cause of lower METC components in RGC-5 cells. We then compared mitochondrial isolates from the different cell types for a non-METC mitochondrial protein, the voltage-dependent anion channel (VDAC). There was a small decrease in VDAC expression in mitochondria isolated from RGC-5 cells in comparison to cerebral and neuroblastoma cells, but not enough to explain the significant difference in complex I expression.

**Figure 6:**
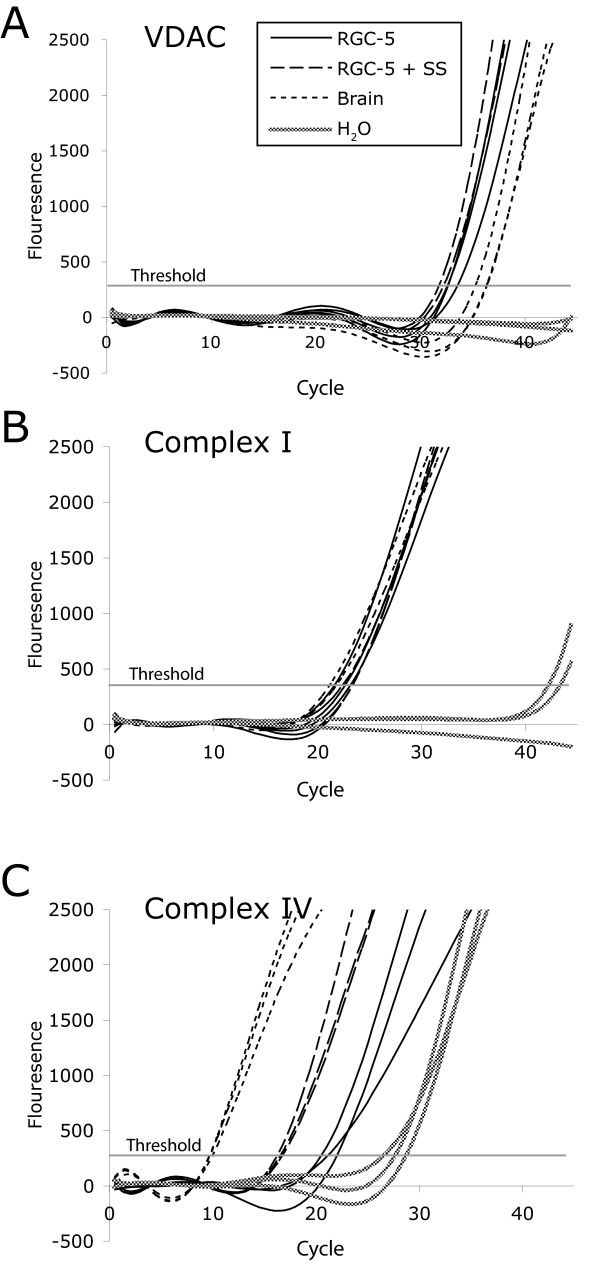
**Quantitative Real-Time RT-PCR of Mitochondrial Components and Associated Proteins**. Amplification curves for (A) VDAC, (B) Complex I, and (C) Complex IV. Solid black lines indicate undifferentiated RGC-5 cells, long dashes indicate differentiated RGC-5 cells, and short dashes indicate cerebral cells. The fuzzy line denotes the template-free control. The horizontal gray line indicates the calculated threshold value by which C_t_ is determined.

We used real-time RT-PCR to compare mRNA levels of mitochondrial electron transport chain complexes I, II, III, IV, V, SOD-2, PMP-70, and VDAC to see if cell type-specific gene transcription levels correlated with protein levels. When normalized to VDAC expression, all METC subunits were downregulated in both undifferentiated and differentiated RGC-5 cells (Table 6). In general, differences in mRNA levels between cell-types correlated with differences in protein levels. Surprisingly, complex I (alpha subunit, 9) transcription levels were higher in brain than RGC-5 cells, but this difference was not seen to the same degree in protein levels, measured by immunoblotting. On the other hand, complex IV (subunit 1) transcription levels were much lower than the difference in protein levels would predict (Figure 6). Differentiation of RGC-5 cells had a minimal effect on transcription levels of all genes studied.

**Table 6:** Relative transcription levels of METC components

Gene	Subunit		RGC-5	RGC-5 +SS
VDAC	-	ΔC_t_	-1.78	-1.73
		Δ(ΔC_t_)	0.0	0.0
		Relative Expression	1.0	1.0
SOD2	-	ΔC_t_	1.57	1.83
		Δ(ΔC_t_)	3.35	3.67
		Relative Expression	0.098	0.08
PMP70	-	ΔC_t_	1.30	1.60
		Δ(ΔC_t_)	3.08	3.33
		Relative Expression	0.118	0.099
C I	NDUFA9	ΔC_t_	0.88	0.67
		Δ(ΔC_t_)	2.67	2.40
		Relative Expression	0.157	0.189
C II	SDHA	ΔC_t_	4.49	3.33
		Δ(ΔC_t_)	6.28	5.07
		Relative Expression	0.013	0.030
C III	UQCRC2	ΔC_t_	3.80	2.92
		Δ(ΔC_t_)	5.58	4.65
		Relative Expression	0.021	0.039
C IV	MTCO1	ΔC_t_	9.58	7.38
		Δ(ΔC_t_)	11.37	9.12
		Relative Expression	0.00038	0.0018
C V	ATP5A1	ΔC_t_	0.48	0.98
		Δ(ΔC_t_)	2.27	2.72
		Relative Expression	0.208	0.152

Changes in transcription levels of each gene studied are given in terms of the difference in threshold cycle compared to cerebral cells(ΔC_t_), the difference in threshold cycle relative to that of VDAC (Δ(ΔC_t_)), and the relative expression level, given as a percent of cerebral and normalized to VDAC expression.

## Discussion

Mitochondria isolated from the RGC-like RGC-5 cell line produce significantly less superoxide than cerebral and neuroblastoma mitochondria when incubated with complex I or II substrates or when treated with complex I or III inhibitors. Correspondingly, RGC-5 mitochondria contain significantly lower levels of complex I compared to other METC components, despite similar levels of transcription. Together, these findings imply that neuronal mitochondria behave differently depending on the cell of origin.

Overall, the rate of superoxide production of RGC-5 cell mitochondria was much less than that of cerebral mitochondria for a given amount of mitochondrial protein. Comparing the rates of production in the absence of substrates and inhibitors, basal cerebral mitochondria superoxide production was seven times of that of RGC-5 cells. The most notable differences after METC inhibition was the increased superoxide production in cerebral mitochondria in the presence of the complex III inhibitor antimycin A. After treatment with antimycin A, cerebral mitochondria had a more than 2-fold increase in superoxide production, compared to an insignificant decrease in superoxide production rate in RGC-5 mitochondria.

The difference in superoxide production is not a result of RGC-5 cells being undifferentiated mitotically active cells. We previously showed that low-dose staurosporine, a broad spectrum protein kinase inhibitor, can induce RGC-5 cells to differentiate to a phenotype that is similar to a mature RGC [14]. To see if differentiation affected ROS production, we compared superoxide production in differentiated and undifferentiated RGC-5 cells. Differentiated and undifferentiated RGC-5 produced similar amounts of superoxide in the presence of complex I substrates and inhibitors and only differed slightly in superoxide production after incubation with succinate, a complex II substrate, in the presence of the complex III inhibitor antimycin A. Differences in superoxide production between mitochondria from RGC-5 cells and cerebral cells were also not due to differences in the source of the tissue (a cell line vs. fresh tissue), because similar differences were seen when RGC-5 cells were compared to a neuroblastoma cell line. Finally, the differences were also not a result of differential purification of mitochondria, as the levels of the peroxisomal-associated protein PMP70 were similar across cell-type.

There are limitations to our study. We did not measure superoxide directly, instead indirectly measuring superoxide generation using the H_2_O_2 _probe Amplex Red. Indirect superoxide measurement with a probe specific for H_2_O_2 _is commonly performed [15,16] because mitochondrial superoxide dismutase (SOD-2) converts superoxide to H_2_O_2_, and the latter freely crosses organelle and cell membranes. Amplex Red reacts with H_2_O_2 _in the presence of HRP and is converted to the fluorescent product resorufin. In these experiments we detected changes in the levels of resorufin and used them to determine the production rates of superoxide. The production of hydrogen peroxide by complex I does not necessarily require dismutation [17]. suggesting that measuring levels of H_2_O_2 _is an acceptable method for measuring superoxide production or leakage from complex I. Nonetheless, we cannot exclude the possibility that abnormally low SOD-2 activity biased our results.

We also examined superoxide production in mitochondria obtained from the RGC-5 cell line, and not from freshly isolated RGCs. It is possible that some of the differences that we measured in superoxide generation arose because the cerebral mitochondria were isolated from whole cerebral tissue and the RGC-5 mitochondria were isolated from a clonal cell line. The environment of the RGC-5 is normoxic, being exposed to atmospheric partial pressure of oxygen, compared to the relatively hypoxic environment within the brain. Also, cerebral mitochondria were isolated from a mixture of cell types, not only neuronal, but also glial cells, and this could blur differences between the cerebral and RGC-5 mitochondria. Purifying a usable quantity of mitochondria from freshly isolated RGCs is impractical, and thus the use of a differentiable RGC cell line allows otherwise unfeasible mitochondrial studies. Difficulties in isolation of mitochondria from RGCs include the large numbers of animals necessary to obtain enough RGCs (there are approximately 10^5 ^RGCs per rat retina) and subsequently obtaining mitochondria from a small number of purified RGCs. It is impractical to do mitochondrial metabolic experiments from freshly isolated RGCs, as discussed above, and whole retina cannot be used because RGCs make up only a small fraction of the tissue.

Low levels of superoxide production in RGCs compared to cerebral and neuroblastoma cells could be due to decreased flow of oxygen through the METC, decreased leakage of superoxide from the METC, or increased scavenging via non-SOD mechanisms (increased scavenging via SOD would increase H_2_O_2 _levels in our assay). Our experimental design could not distinguish these possibilities. The explanation is not simply one of lower METC density in RGCs. Immunoblotting demonstrated that there was markedly less mitochondrial complex I in RGC-5 cells compared to cerebral and neuroblastoma cells, while levels of complexes II, III, and IV were similar. Analysis of transcription levels confirms lowered gene expression of these complexes in RGC-5 cells compared to cerebral cells. If there were also lower amounts of other METC components in RGC-5 cells, then this could explain why RGC-5 mitochondria produce less superoxide. However, if the differences in superoxide were simply a matter of lower concentration of METC components, then this could explain differences in baseline superoxide production, but would not explain differences in patterns of superoxide induction, e.g. why there was such a large increase in superoxide production in cerebral mitochondria but not RGC-5 cells after treatment with antimycin A. It has been shown that superoxide production by isolated succinate dehydrogenase is highly dependent on succinate concentration, with superoxide generation increasing, then decreasing with succinate concentration [18]. Lower expression of succinate dehydrogenase and other METC components, as suggested by our immunoblotting and RT-PCR data, may lead to succinate blocking autooxidation at a lower concentration in RGC-5 cells compared to cerebral cells. Another possibility relates to the fact that antimycin A leads to increased superoxide production by backflow of electrons into complex I. If complex I levels are lower in RGC-5 cells, then this would lead to less superoxide production after antimycin A treatment in those cells.

We speculate that decreased production of superoxide by RGCs may have a physiological rationale. We previously demonstrated RGCs use superoxide as a mitochondrial-derived intracellular messenger for signaling the initiation of apoptosis after axonal injury [10]. If RGCs use superoxide to signal cell death, then it is reasonable to presume that they also tightly regulate its intracellular concentration in relevant compartments. Otherwise there would be death of a critical (and non-renewable) class of neurons.

Loss of this tight regulation of superoxide production could also explain the mechanism of RGC death from mtDNA mutations in LHON. Although it is commonly believed that LHON results from RGCs having high-energy requirements, with mtDNA mutations causing RGC-specific death because the cell cannot meet its energy demands. However, this theory does not clearly address why there is little neuronal loss in other high-energy cells such as photoreceptors or cardiac muscle (as is seen in some mtDNA deletions), nor does it explain the delayed onset of LHON. Most importantly, there is a discrepancy between the degree of ATP deficiency and the severity of the optic neuropathy in cells with LHON mutations [19].

Instead, it is more likely that differences in ROS production and sensitivity to oxidative stress among cells types explains the timing and the specificity of LHON. There are precedents for cell type-specific differences in mitochondrial superoxide production. Rat brain and liver mitochondria differ in superoxide production in the presence of METC substrates and inhibitors [20]. Cardiac mitochondria from birds and mice have dramatically different rates and sites of H_2_O_2 _production [21]. Finally, LHON cybrids in neuronal-like differentiated NT2 cells have greatly increased superoxide production [6]. It makes sense that ROS would be involved in LHON because the mutations are in known superoxide production sites [22], inhibition of these sites dramatically increases superoxide synthesis [15], and neuronal cybrids with LHON mutations have increased superoxide production [6]. In cerebral mitochondria, complex I produces the majority of superoxide radicals [16], and the same may be true for RGCs [10]. If RGC mitochondria have insufficient mechanisms for reducing levels of superoxide production, particularly in response to METC inhibition, then mutations of critical complex I components could theoretically lead to catastrophic superoxide production and anomalous signaling of cell death in the absence of axonal damage [10]. Lower METC component expression and basal superoxide production in RGCs may be accompanied by a similar downregulation in SOD2 or other ROS-reducing systems, leading to a phenotype which is overwhelmed more readily than other cells in the event of aberrant superoxide generation.

## Conclusion

Mitochondria from a RGC-like cell line produced superoxide at a much lower rate than cerebral or neuroblastoma mitochondria, and there was a dramatic difference in superoxide production when electrons were shunted to complex I using METC complex I and III substrates and inhibitors. These differences were mirrored by different patterns of METC component expression between cell types. Decreased superoxide production may be essential for preventing aberrant signaling of cell death in RGCs. Mutations coding for specific components of NADH-ubiquinone oxidoreductase in LHON may disrupt the cell type-specific handling of superoxide in RGC mitochondria and lead to premature cell death. This could explain why LHON mtDNA mutations are present in all cells, but the clinical phenotype is predominantly one of RGC death.

## Methods

### Animals

Cerebral mitochondria were obtained from adult Long-Evans rats. All animals were used in accordance with federal, state, and institutional guidelines for the use of animals in laboratory research.

### Materials

Staurosporine (from *Streptomyces staurosporeus*; ≥ 98% purity; catalog number 380-014) was obtained from Alexis Biochemicals (San Diego, CA. Cell culture reagents, unless noted, were obtained from BioWhittaker (Rockland, ME). All labeled antibodies and fluorescent dyes were obtained from Molecular Probes (Eugene, OR), Sigma (St. Louis, MO), Jackson ImmunoResearch Laboratories (West Grove, PA), and Abcam (Cambridge, MA). Rotenone and 3-(4,5-dimethylsulfoxide (DMSO) was obtained from Sigma (St. Louis, MO). Antimycin A was from Fisher Scientific (Hampton, NH). Mitochondria isolation materials and protease inhibitors were from Pierce Biotechnology (Rockford, IL).

### Solutions

Amplex Red solution consisted of 100 μM Amplex Red reagent, 0.2 U/ml horseradish peroxidase (HRP), and 0.05 M sodium phosphate. Mannitol, tris-HCl, and potassium chloride (MTP) solution consisted of 110 mM mannitol, 60 mM Tris-HCl, 60 mM KCl, 10 mM KH_2_PO_4_, 0.5 mM EDTA, pH 7.4.

### Cell Culture and Differentiation

The RGC-5 cell line was generously provided by Dr. Neeraj Agarwal of the University of North Texas. RGC-5 cells were cultured in DMEM (Mediatech, Inc.; Herndon, CA) containing 1 gm/L glucose with L-glutamine, supplemented with 10% fetal bovine serum, 100 U/ml penicillin, and 100 μg/ml streptomycin. Cells were incubated at 37°C in humidified 5% CO_2_. To induce differentiation some RGC-5 were treated with staurosporine (316 nM) 24 hours prior to mitochondria isolation. The neuroblastoma SK-N-AS line was obtained from Dr. Arthur Polans of the University of Wisconsin (Madison). Cells were cultured in RPMI 1640, supplemented with 10% fetal bovine serum, 10 mM HEPES, penicillin 100 U/ml, streptomycin 100 μg/ml, and amphotericin B 0.25 μg/ml.

### Isolation of RGC-5 and Neuroblastoma SK-N-AS Mitochondria

Cells were treated with trypsin-EDTA to allow detachment from growing flasks, centrifuged 7 minutes at 250 × g at 4°C. Mitochondria were isolated from the pellet using the Pierce Mitochondria Isolation Kit for Mammalian Cells (Pierce Biotechnology, Rockford, IL, Prod # 89874) using the reagent-based protocol. Briefly, cells were treated with reagents in the presence of Pierce Halt Protease Inhibitor Cocktail, EDTA free (Prod # 78415) and were subject to a series of graded centrifugations. To maintain the integrity of the mitochondria, samples were kept on ice during the isolation process. The mitochondrial pellet was suspended in MTP medium and remained on ice until analysis of mitochondrial activity was performed.

### Isolation of Cerebral Mitochondria

Long-Evans rats were euthanized with CO_2 _gas and approximately 100–200 mg of the frontal cortex removed. The tissue was washed with ice-cold PBS and a mitochondrial isolation kit for soft tissues (Pierce, Prod # 89801) was used. Briefly, the brain tissue was cut into small pieces and added to a cold Dounce homogenizer containing PBS. The tissue was homogenized using 7–10 strokes of the homogenizer. The homogenate was then centrifuged per protocol and the resultant mitochondrial pellet suspended in MTP and kept on ice until analyzed.

### Protein Content of Mitochondrial Preparations

Isolated mitochondria pellets were suspended in MTP and the protein content of the isolated mitochondria determined by Bradford assay. For each experiment protein concentrations were adjusted so that each mitochondrial sample had the same protein content.

### MitoTracker Green FM Quantification of Mitochondrial Preparations

The fluorophore MitoTracker Green FM was used as a secondary method to verify that the mitochondrial content was similar between cell types. MitoTracker Green FM becomes a fluorescent thiol-conjugate (excitation 490 nm/emission 515 nm) after oxidation in the lipid membrane of mitochondria. Solutions containing mitochondria were treated with MitoTracker Green FM (0.5 μM) in a 1:1 ratio and were allowed to aggregate fluorescent product for 30 minutes in a 96-well plate. Fluorescence was then compared between cell type mitochondria using a Wallac Victor^2 ^1420 multilabel counter with appropriate filters.

### Mitochondrial Electron Transport Chain Content of Mitochondrial Preparations

Mitochondrial enriched and depleted (i.e. cytoplasm) samples obtained from the final supernatant of the mitochondria isolation procedure were stored in NuPage LDS sample buffer with reducing agent (Invitrogen). The protein concentrations of the isolated mitochondria were determined by Bradford assay, and equal amounts of protein for each mitochondrial preparation were boiled in the presence of 4× lithium dodecyl sulfate (LDS) sample buffer (Invitrogen, Carlsbad, CA) plus 5% β-mercaptoethanol, resolved on a Bis-Tris 4% to 12% polyacrylamide gel (NuPAGE; Invitrogen), and transferred overnight at 50 mA to nitrocellulose membrane in a transfer apparatus (Mini Protean II; Bio-Rad Laboratories, Hercules, CA). After transfer, the membrane was blocked with 0.5% nonfat milk in TBS (pH 8.0) for 30 minutes and then individually probed with mouse monoclonal antibody to NADH:ubiquinone oxidoreductase α subcomplex, 9 (complex I; 1:2,000; Molecular Probes, Eugene, OR), mouse monoclonal antibody to succinate dehydrogenase flavoprotein (complex II; 1:2,500; Molecular Probes, Eugene, OR), mouse monoclonal antibody to ubiquinone:cytochrome c oxidoreductase core III (complex III; Molecular Probes, Eugene, OR), mouse monoclonal antibody to cytochrome C oxidase subunit I (complex IV; 1:1000; Molecular Probes, Eugene, OR), rabbit monoclonal antibody to voltage dependent anion channel 1/porin (VDAC; 1:500; Sigma, St. Louis, MO), or rabbit monoclonal antibody to PMP70 (1:2000; Abcam, Cambridge, MA). Blots were rinsed three times with TBS containing 0.05% Tween-20 (Fisher Scientific), then washed 5 times for 10 minutes each at room temperature on an orbital shaker. Secondary antibodies used were horseradish peroxidase (HRP)-conjugated goat anti-mouse IgG (1:5000; Jackson ImmunoResearch Laboratories, West Grove, PA) or purified horseradish peroxidase (HRP)-conjugated goat anti-rabbit IgG (1:5000; Jackson ImmunoResearch Laboratories, West Grove, PA) and were incubated for 1 hour at room temperature, followed by 3 rinses and five 10-minute washes with TBS containing Tween-20 at room temperature on an orbital shaker. Blots were treated with freshly prepared ECL solution containing 100 mM Tris-HCl (pH 8.5), 1.25 mM luminol, 225 μM *p*-coumaric acid (Sigma-Aldrich), and 1 mM H_2_O_2 _Fisher Scientific) for 1 minute, and excess solution was allowed to drip off. The blots were then exposed to film (BioMax XAR; Eastman Kodak Company, Rochester) and developed. The films were scanned at 1600 dpi and band density was determined by comparing total intensity in an area containing the band of interest to the intensity of an equal size area of background using NIH ImageJ software. Band density readings are presented with respect to the density of the band from cerebral mitochondria.

### RT-PCR Analysis of Expression of Components of the Mitochondrial Electron Transport Chain

Total RNA was isolated from 0.5–1.0 × 10^6 ^RGC-5 cells, differentiated RGC-5 cells, and rat brain using the RNeasy Protect Mini Kit (Qiagen; Valencia, CA). Freshly prepared RNA samples were immediately transcribed to cDNA using the iScript cDNA Synthesis Kit (Bio-Rad; Hercules, CA) and quantitative real-time PCR was carried out with primers to each gene subunit analyzed by Western blotting (Table 7) using the iScript One-Step RT-PCR Kit with SYBR Green, omitting reverse transcriptase. Primers were either taken from previously published papers or designed using Primer Express (Applied Biosystems; Foster City, CA). Specificity was confirmed by searching against rat genomic and expression nucleotide databases. Samples were cycled 45 times for 15 sec at 95°C and 30 sec at 55°C, followed by 1 min at 95°C and 1 min at 55°C. Melt curve analyses were performed, and single peaks observed in all cases.

**Table 7:** Primers Sequences used for RT-PCR Analysis and Expected Product Sizes

Gene	Subunit	Primer Sequence 5'-3'	PCR Product
SOD2	-	F: ATT AAC GCG CAG ATC ATG CA	89 bp
		R: TCG TGG TAC TTC TCC TCG GTG A	
VDAC	-	F: ATG CCT GCT TTT CGG CCA A	118 bp
		R: GCT GGA TGG CAA GAA CGT CAA	
PMP70	-	F: CTC GGC CTG CAC GGT AAG	78 bp
		R: CAC AGC TCG CTC TTT CTT TCC T	
C I	NDUFA9	F: TTG TCA ACC ATC TTG GAC GAA T	166 bp
		R: TTG CTG TGC TGC ACT GCT TTC	
C II	SDHA	F: GAG GAA GCA CAC CCT CTC ATA	101 bp
		R: TAG CAC AGT CAG CCT CAT TCA A	
C III	UQCRC2	F: AAA TTG ACG CTG TGG CTG ATG	101 bp
		R: AGG CGT ATG TCC CAA GTT TCC	
C IV	MTCO1	R: AGC CCC TGA TAT AGC ATT CCC	116 bp
		F: TTC ATC CTG TTC CAG CTC CAG	
C V	ATP5A1	F: CTT ATC CAA GCA GGC TGT TGC	134 bp
		R: AAT CGT TCA TCT TGG CTG CTC	

Threshold cycle (C_t_) was computed automatically by the iCycler software (Bio-Rad), and compared across all conditions. Delta C_t _(ΔC_t_) was computed for each gene with respect to brain for each cell line studied, and normalized for VDAC expression by subtracting the ΔC_t _for VDAC in a particular cell type from the ΔC_t _for each other gene in that cell type (Δ(ΔC_t_)). This difference indicates the -(log2) difference in RNA copy number.

### Superoxide Measurements

Superoxide was indirectly measured by assaying H_2_O_2 _[15,16], the product of superoxide dismutation by mitochondrial SOD-2. The fluorescent product of Amplex Red and H_2_O_2 _was analyzed in various conditions in duplicate to quadruplicate. All assays were performed in 96-well plates and read with a Wallac Victor^2 ^1420 multilabel counter using the appropriate filters (excitation 535 nm/emission 580 nm). To each well Amplex red solution was added and a time-lapse fluorescent baseline was established in the absence of mitochondria. Consecutively, to the well containing Amplex red was added an equal volume of cerebral, RGC-5, or neuroblastoma mitochondria, a substrate (glutamate/malate or succinate), and an inhibitor of the METC (rotenone or antimycin A). All figures are expressed in nmol/min/mg protein, assuming full superoxide dismutation. All experiments were repeated 2–4 times.

### Statistical Analysis

Comparisons between different groups were by Student's unpaired t-test, while comparisons between serial treatments of mitochondria with substrate or inhibitors were by Student's paired t-test. Significant differences required p < 0.05.

## Authors' contributions

MH performed the cell culture, mitochondrial isolations, measurements of superoxide production, and immunoblotting. CL performed the quantitative PCR. LL conceived of the study and designed the experiments. All authors participated in writing and editing the manuscript, read, and approved the final manuscript.
